# Expression of lamin C2 in mammalian oocytes

**DOI:** 10.1371/journal.pone.0229781

**Published:** 2020-04-28

**Authors:** Marketa Koncicka, Jakub Cervenka, Daniel Jahn, Rita Sucha, Petr Vodicka, Ahmed Gad, Manfred Alsheimer, Andrej Susor

**Affiliations:** 1 Laboratory of Biochemistry and Molecular Biology of Germ Cells, Institute of Animal Physiology and Genetics of the Czech Academy of Sciences, Libechov, Czech Republic; 2 Laboratory of Applied Proteome Analyses, Institute of Animal Physiology and Genetics of the Czech Academy of Sciences, Libechov, Czech Republic; 3 Department of Cell and Developmental Biology, Biocenter, University of Würzburg, Würzburg, Germany; 4 Laboratory of Developmental Biology, Institute of Animal Physiology and Genetics of the Czech Academy of Sciences, Libechov, Czech Republic; 5 Department of Animal Production, Faculty of Agriculture, Cairo University, Giza, Egypt; University of Pittsburgh, UNITED STATES

## Abstract

Lamin C2 (LMN C2) is a short product of the *lamin a* gene. It is a germ cell-specific lamin and has been extensively studied in male germ cells. In this study, we focussed on the expression and localization of LMN C2 in fully-grown germinal vesicle (GV) oocytes. We detected LMN C2 in the fully-grown germinal vesicle oocytes of various mammalian species with confirmation done by immunoblotting the wild type and *Lmnc2* gene deleted testes. Expression of LMN C2 tagged with GFP showed localization of LMN C2 to the nuclear membrane of the oocyte. Moreover, the LMN C2 protein notably disappeared after nuclear envelope breakdown (NEBD) and the expression of LMN C2 was significantly reduced in the oocytes from aged females and ceased altogether during meiotic maturation. These results provide new insights regarding LMN C2 expression in the oocytes of various mammalian species.

## Introduction

Based on biochemical classification and molecular composition, nuclear lamins belong to the type V group of intermediate filament proteins [1,2]. Lamins are components of nuclear lamina and are localized at the boundary of the inner nuclear membrane and the chromatin [3]. In mammals lamins are coded by three genes: *lamin a*, *lamin b1* and *lamin b2*. The *lamin b1* gene encodes lamin B1 protein, while the *lamin b2* gene encodes lamin B2 and the short germ cell specific lamin B3 protein [4,5]. Alternative splicing of *lamin a* produces lamin A, lamin C, lamin AΔ10 and germ cell specific lamin C2 [6–8].

It is known that mammalian spermatocytes do not express lamins A, C and B2 during meiotic prophase I. Only lamins B1 and C2, with 52 kDa the smallest product of the *lamin a* gene, are detected in spermatocytes at this time point of germ cell development [4,7,9–11]. By genomic analysis it was found that mouse LMN C2 for the most part is very similar to LMN C but lacks 112 amino acids from the N-terminal domain which is replaced by the specific hexapeptide GNAEGR. Lamin C2 lacks a CaaX box, which is in somatic lamins important for association with the inner nuclear membrane [12]. The localization pattern of lamin C2 is remarkably different to that of other nuclear envelope proteins in the same cellular context, e.g. lamin B1 and lamin associated proteins 2 (LAPs2), as it does not show continuous rim-like spreading around the nucleus. Instead, lamin C2 forms intermittent domains within the nuclear envelope and is found enriched at telomere attachment sites. In early prophase the telomeres of the chromosomes permanently bind to these LMN C2 enriched sites and subsequently move along the nuclear envelope [13]. [14] generated a knockout mouse model specifically for LMN C2 through the elimination of the lamin C2 specific exon 1a. All other regions of *Lmna* gene were kept intact and consequently the expression of LMN A and LMN C was found to be normal. LMN C2 knockout males were viable but completely infertile. Contrary to the male situation, *Lmnc2*^*-/-*^ females were fertile and produced offspring. *Lmnc2*^*-/-*^ oocytes and spermatocytes both show defects in synaptic pairing but in the spermatocytes the defects were significantly worse than in the oocytes. *Lmnc2*^*-/-*^ spermatocytes in mid-pachytene undergo apoptosis before completing double-strand break repair (DSB) and crossing over. Oocytes have reduced meiotic recombination rates but almost half of the chromosomes are able to make valid crossing over. Although as yet not analysed, it is tempting to speculate that a decreasing meiotic recombination and cross over rate in the *Lmnc2*^*-/-*^ oocytes could finally result in chromosomal segregation defects that will become overt during the anaphase I stage of meiotic progression [14].

To address this issue, in the present study we performed a detailed analysis of LMN A/C expression in oocytes. We show that LMN C2 is expressed in fully grown mouse oocytes and disappears during meiotic progression. These results reveal that lamin C2 is not just male germ cell specific.

## Results

### Lamin C2 is expressed in the mouse oocyte

**Fig 1:**
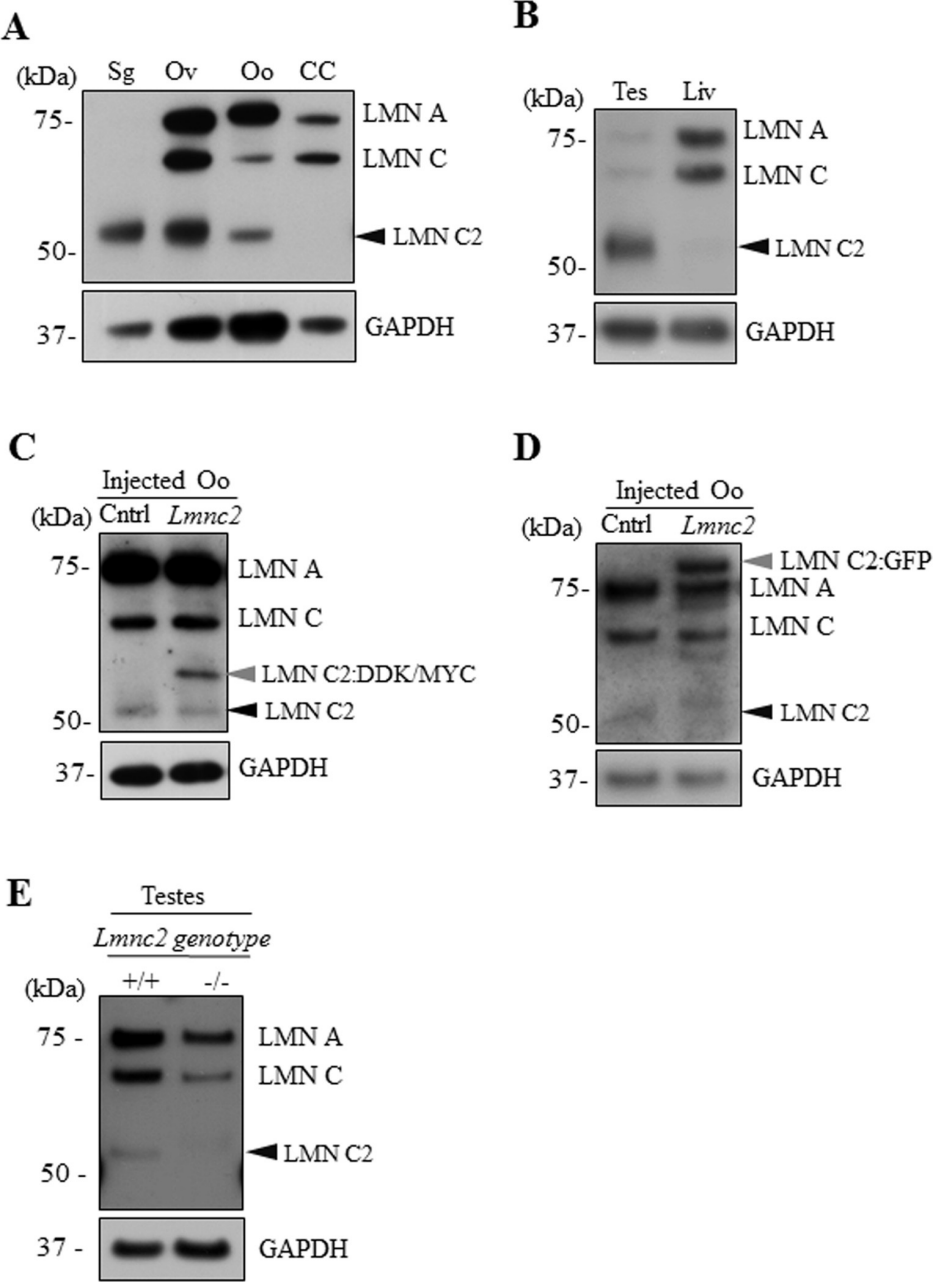
Lamin C2 is expressed in oocyte, ovary and testes but not in cumulus cells. A) Representative immunoblot image showing expression of LMN A/C isoforms in mouse spermatogenic cells (Sg), ovary (Ov), oocytes (Oo) and cumulus cells (CC). Black arrowhead depicts endogenous LMN C2 protein. B) Representative immunoblot image showing expression of LMN A/C isoforms in testes (Tes) and liver (Liv). Black arrowhead depicts endogenous LMN C2 protein. C) Microinjection of expression plasmid coding for LMN C2:DDK/MYC shows expression of additional band (grey arrowhead) above endogenous LMN C2 protein (black arrowhead). Non-injected oocytes were used as a control (Cntr). D) Expression of LMN C2 tagged with GFP (arrowhead) in plasmid microinjected oocytes. Black arrowhead depicts endogenous LMN C2 protein. E) For validation of the LMN A/C antibody we used testicular samples with +/+ and -/- genotype of genomic *Lmnc2* allele. Black arrowhead depicts endogenous LMN C2 protein. GAPDH was used as an endogenous loading control. Representative images from at least three independent experiments are shown.

LMN C2 was previously detected in spermatogenic cells [7,9,10,15,16] and later in embryonic oocytes [14]. Here we asked if LMN C2 is also expressed in the fully-grown mammalian oocyte. On immunoblot membranes with germ cell samples and selected somatic cell controls, pan-antibody against LMN A/C detects LMN C2 (52 kDa) as a dominant product of the *lmna* gene only in the spermatogenic cells (Sg). All the lamin A variants, LMN A (70 kDa), C (60 kDa) and C2 (52 kDa) were observed in the ovary (Ov) and mouse oocytes (Oo), while cumulus cells (CC) isolated from cumulus oocyte complexes only included the somatic LMN A (70 kDa) and C (60 kDa) isoforms (Fig 1A). To ensure that the 52 kDa signal indeed corresponds to LMN C2 and not to a previously described 55 kDa LMN A/C degradation product [17], we tested the pan-antibody LMN A/C on testes and liver samples. As expected, the antibody used for immunoblot detection recognized the 52 kDa protein in the testis but not in the liver sample, indicating that the 52 kDa signal does represent LMN C2 (Fig 1B). Besides the high level of LMN C2 in the testicular sample, it also shows a low amount of LMN A and C (Fig 1B) which is likely due to the presence of few somatic cells in the testicular tissue sample.

The lamin A/C antibody was validated by microinjecting an expression vector coding for LMN C2 tagged with DDK/MYC into transcriptionally active germinal vesicle (GV) oocytes. Immunoblotting showed the expression of exogenous LMN C2:DDK/MYC protein, which is seen above the endogenous LMN C2 band in the oocytes injected with the plasmid (Fig 1C). Injections of RNA coding for LMN C2 tagged with GFP [12] into oocytes show an extra protein band at the 80 kDa level which corresponds to LMN C2:GFP (LMN C2–52 kDa and GFP– 27 kDa) (Fig 1D). Additional faint bands in the immunoblots of oocytes microinjected with *Lmnc2*:*gfp* RNA, just below the Lamin A and Lamin C, are most likely result of degradation of ectopically expressed LMN C2:GFP (Fig 1D). Next, we validated the LMN A/C antibody using protein lysates from *Lmnc2*^*+/+*^ mouse testes and testes of mouse with completely deleted *Lmnc2*^*-/-*^. As expected, the immunoblot displayed a strong band corresponding to the LMN C2 protein in the *Lmnc2*^*+/+*^ (Fig 1E), while the testicular sample from the *Lmnc2*^*-/-*^ animal showed a complete absence of the LMN C2 signal (Fig 1E; for comparison see [14].

In conclusion, our results show that the LMN C2 protein is expressed in fully-grown mouse oocytes.

### LMN C2 is localized at the nuclear membrane of mouse oocytes

**Fig 2:**
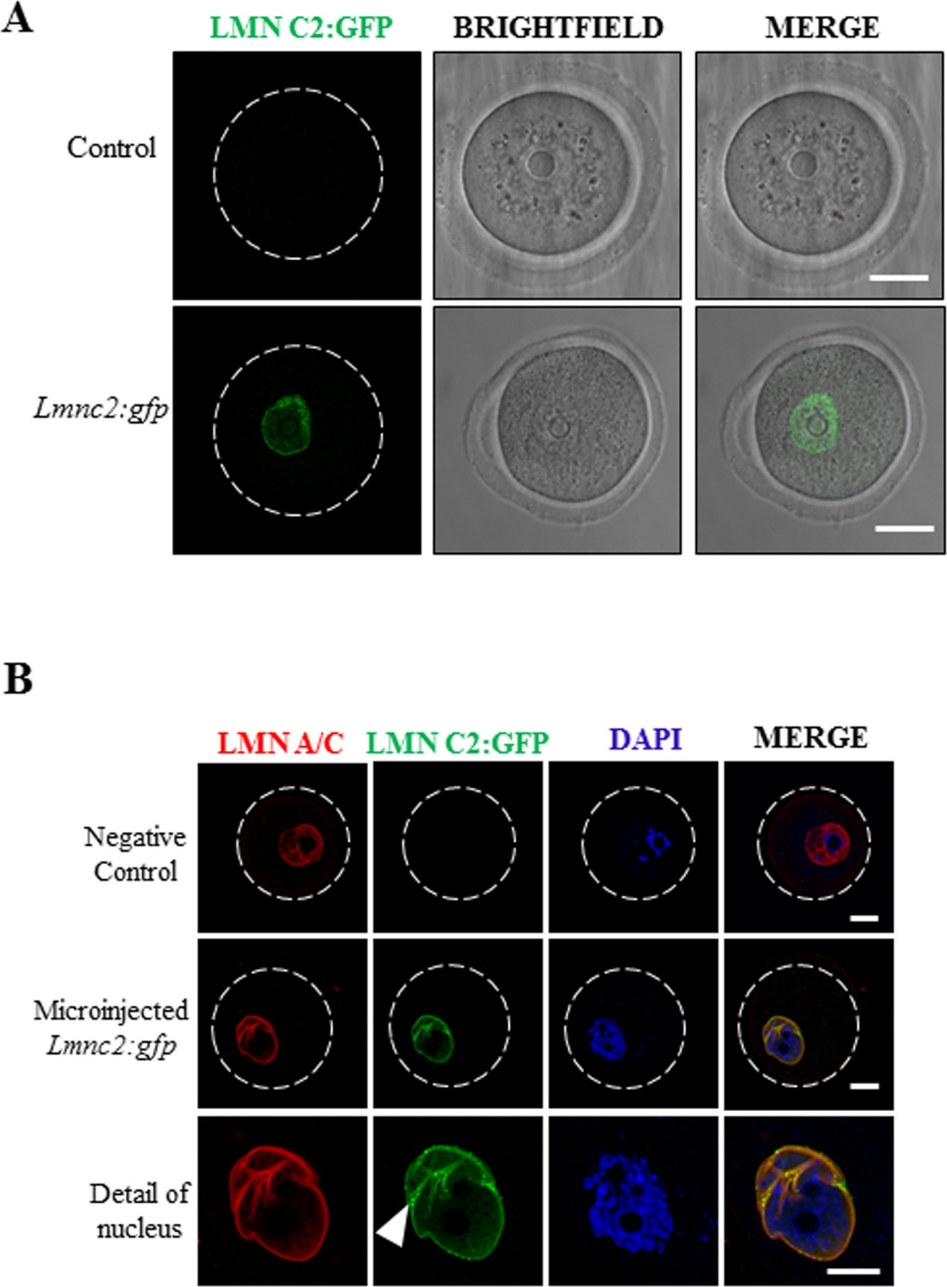
LMN C2 is localized at the nuclear membrane in mouse oocytes. A) Expression and localization of LMN C2:GFP in living oocytes. Scale bar, 25μm. B) Expression of LMN C2:GFP (green) and immunocytochemical detection of endogenous lamin A/C using pan-LMN A/C antibody (red). DNA stained by DAPI (blue). White arrowhead depict LMN C2:GFP puncta. Scale bar, 10 μm. Representative images from at least three independent experiments are shown. See also Fig 1C.

Next we asked where in the mouse oocyte is LMN C2 specifically localized. We carried out the expression of LMN C2 tagged with GFP in the GV oocyte. Transcriptionally active mouse oocytes were injected with expression plasmids coding for LMN C2:GFP [12] and live oocytes were imaged for LMN C2:GFP fusion protein (Fig 2A).

Oocytes expressing LMN C2:GFP were then immunostained using LMN A/C antibodies (Fig 2B, red channel). Imaging showed the distinct expression of exogenous LMN C2:GFP (Fig 2B, green channel) demonstrating a punctate pattern at the nuclear envelope. The LMN A/C antibody (red), which detects both the ectopically expressed LMN C2:GFP and the endogenous A type lamins, revealed colocalization with the GFP signal at the nuclear envelope (Fig 2B). As expected, oocytes that do not express LMN C2:GFP do not produce a green fluorescence signal (negative control) (Fig 2B).

### Oocytes from *Lmnc2*^*-/-*^ animals express A-type lamins and do not exhibit morphological abnormalities

**Fig 3:**
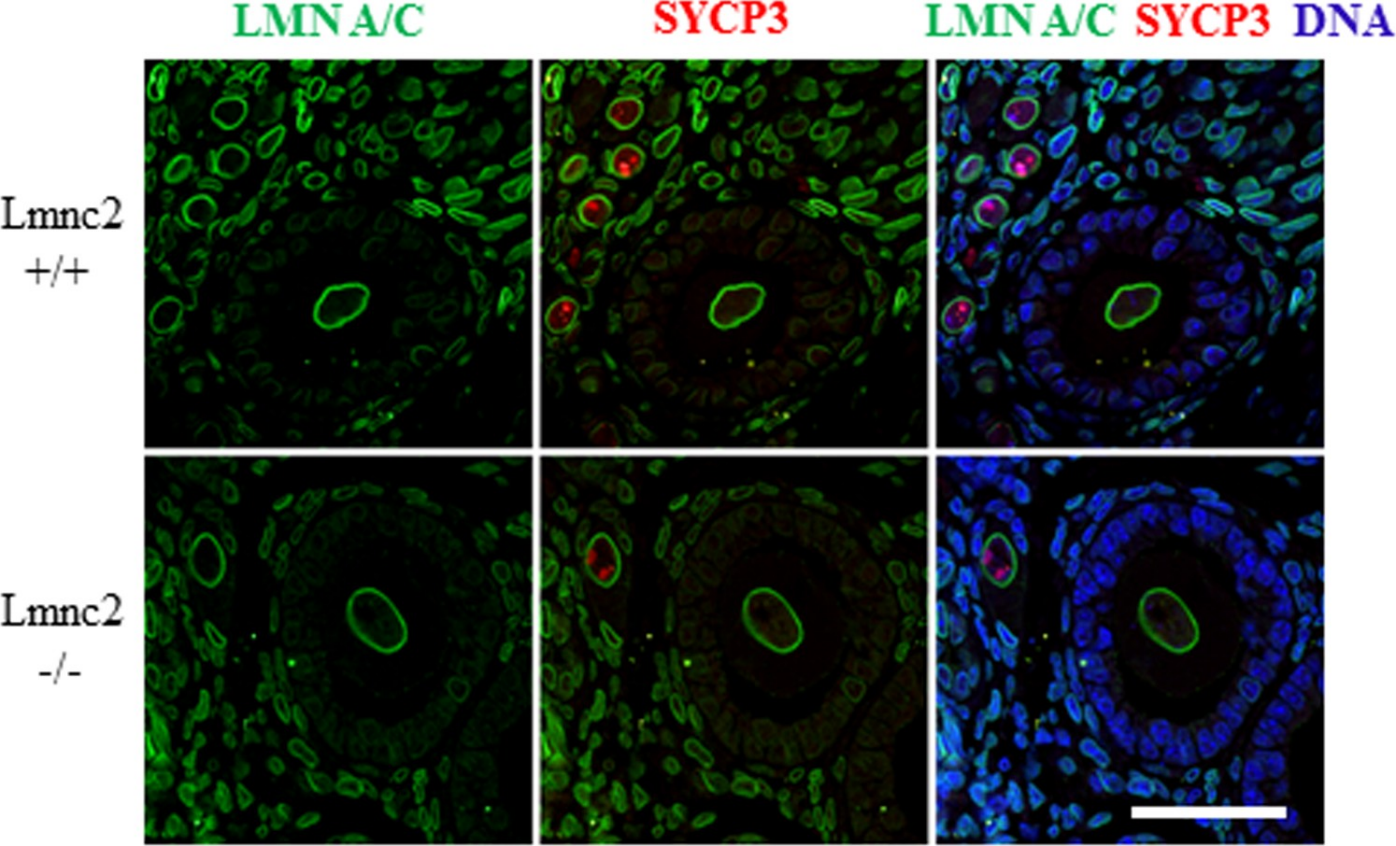
Oocytes from *Lmnc2*^*-/-*^ animals express A-type lamins and do not exhibit morphological abnormalities. Immunohistochemistry on 3 μm paraffin sections of ovaries from 11-day old females of *Lmnc2*^*+/+*^ (upper row) and *Lmnc2*^*-/-*^ (lower row) animals labeled with LMN A/C (green) and synaptonemal complex protein (SYCP3; red) antibodies. DNA is counterstained with Hoechst 33258 (blue); scale bar, 40 μm.

Using slices of ovaries isolated from *Lmnc2*^*+/+*^ and *Lmnc2*^*-/-*^ mice we next asked whether in the absence of LMN C2 follicular morphology is impaired (Fig 3). 3 μm ovary sections from 11d postpartum wild type and *Lmnc2*^*-/-*^ females were stained with LMN A/C antibody (#bs-01, [17]) and the meiotic prophase marker synaptonemal complex protein 3 (SYCP3). As expected, this staining produced typical lamin A/C signals in the somatic cells of the ovaries of both the wild type and the *Lmnc2*^*-/-*^ females. Besides this, the LMN A/C antibody also strongly labelled the nuclear membrane of the growing oocytes without any difference between the WT and the *Lmnc2*^*-/-*^ females (Fig 3). This finding is consistent with our immunoblot data on fully grown oocytes of wild type mice (Fig 1A) and thus affirms that the postnatal oocytes express LMN A and LMN C in addition to LMN C2.

The ovary sections were also analysed for any morphological defects in the ovarian follicles. As seen in (Fig 3), oocytes of the 11d old *Lmnc2*^*-/-*^ females appear quite normal and show no overt difference to the corresponding oocytes of the wild type littermates, indicating that LMN C2 deficiency has no effect on general follicular development and growth. Also, the nuclear shape of the growing oocytes and the distribution of LMN A and LMN C appear to be normal in the LMN C2 deficient background, suggesting that absence of LMN C2 has no or only a very minor influence on the nuclear integrity of the growing oocytes.

### LMN C2 is present only in protein form in the oocyte

**Fig 4:**
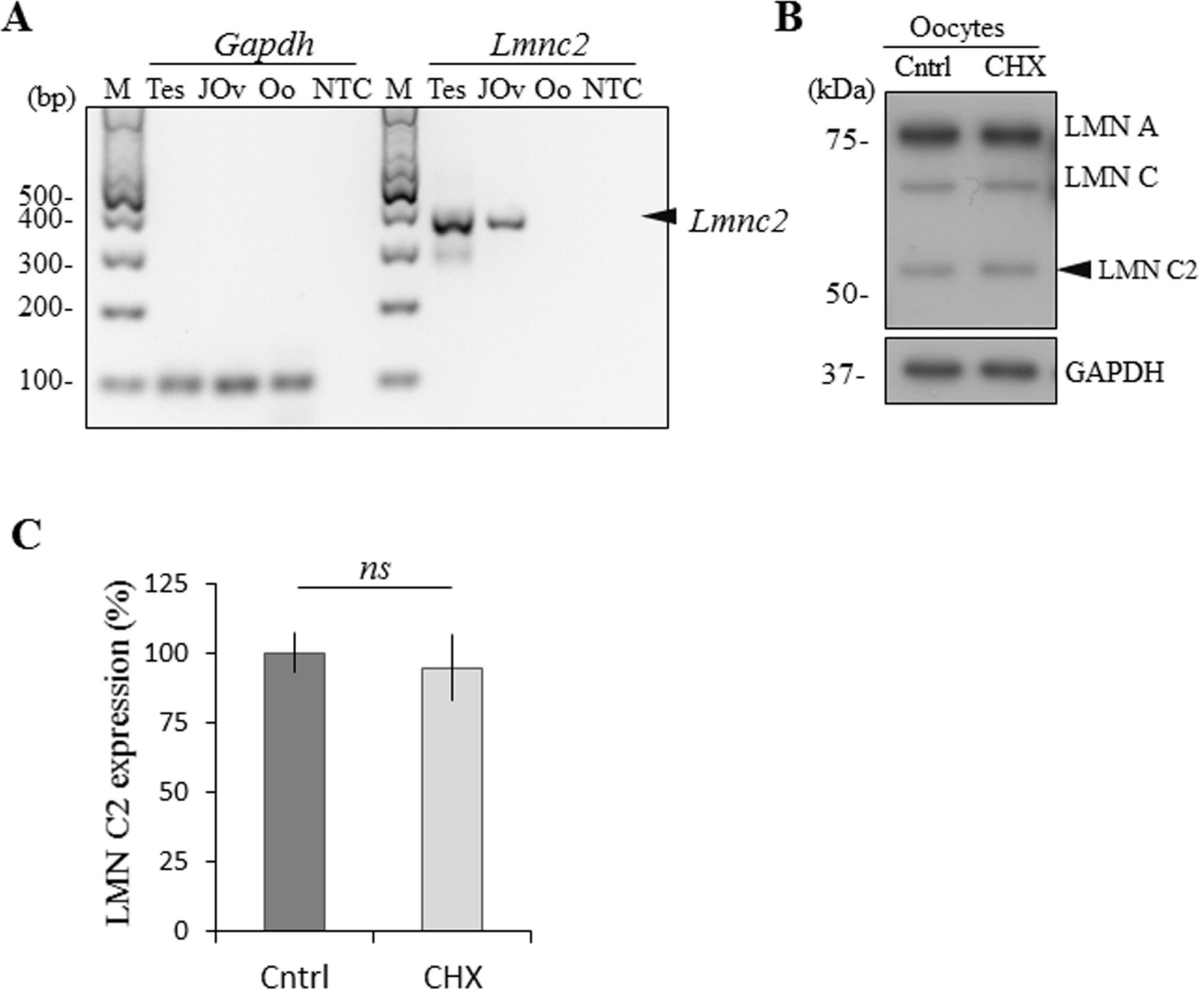
LMN C2 is expressed only in protein form in the oocyte. A) RT-PCR analysis of the *Lmnc2* mRNA expression in the mouse testes (Tes), juvenile ovaries (1-day old mouse pup; JOv) and fully-grown GV oocytes (Oo). GAPDH mRNA was used as a reference gene and reaction without cDNA template was used as a non-template control (NTC), M– 100 bp marker. Black arrowhead depicts endogenous *Lmn c2* mRNA. B) Immunoblot detection of expression of LMN C2 in oocytes treated by translational repressor cycloheximide (CHX). GAPDH was used as an endogenous loading control. Representative images from at least three independent experiments are shown. Black arrowhead depicts endogenous LMN C2 protein. C) Quantification of the expression of LMN C2 protein in oocytes treated by CHX. Normalized to GAPDH, presented as mean, ±SD, ns-nonsignificant, t-test.

Additionally, to confirm and augment our previous results, we performed RT-PCR to detect mRNA coding for LMN C2 in testes and juvenile ovaries in 1 day old mice and in fully grown GV oocytes. We found that *Gapdh* mRNA was present in all samples with almost the same expression levels. However, we detected *Lmnc2* mRNA in the testicular and juvenile ovarian samples, but not in the fully grown GV oocytes (Fig 4A). Next we validated our RT-PCR results by treating fully grown GV oocytes with the translational repressor cycloheximide (CHX) [18–22]. LMN C2 protein levels were unaffected by inhibition of translation (Fig 4B and 4C), indicating stability of the LMN C2 protein at this oocyte development stage and independence of LMN C2 protein level on new translation. This explains detection of LMN C2 protein in the absence of *Lmnc2* mRNA (Fig 4A). To validate translational repression by CHX we treated GV oocytes by 10 μg/ml for 2hrs in the presence of global translational marker ^35^S-Methionine (S1 Fig).

Our results show that fully grown oocytes express LMN C2 only in the protein form which has been translated earlier during the oocyte growth phase.

### Expression of LMN C2 significantly differs between oocytes from young and aged females

**Fig 5:**
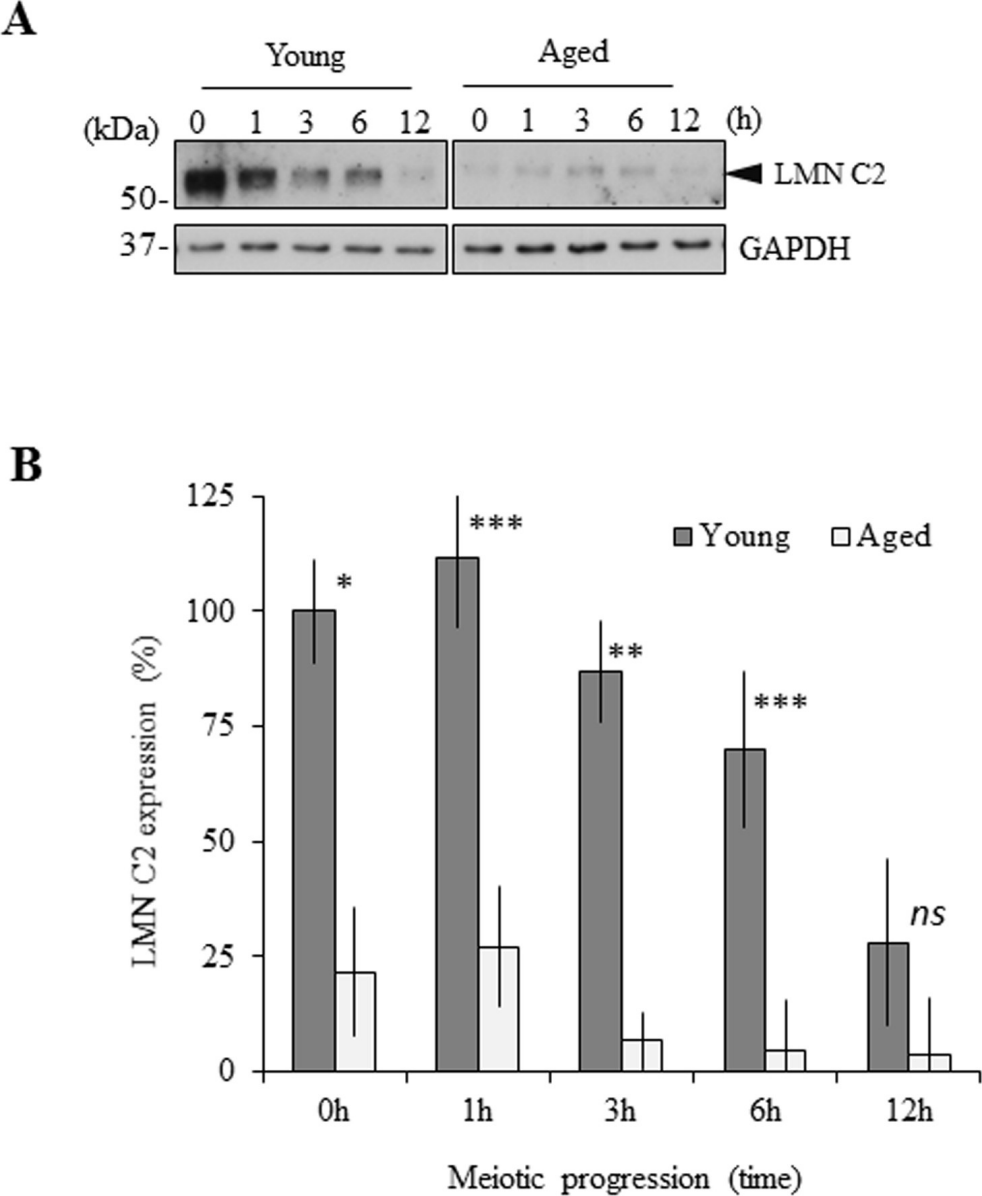
Expression of LMN C2 significantly differs between oocytes from young and aged females. A) Immunoblot detection of LMN C2 and GAPDH protein expression in the oocytes from young (2 months) and aged (12 months) females during meiotic progression. Representative images from at least three independent experiments are shown. Black arrowhead depicts endogenous LMN C2 protein. B) Quantification of LMN C2 expression in the oocytes from young and aged females during meiotic progression. Presented as mean, ±SD, *p<0.05; **p<0.01; ***p<0.001; *ns*-nonsignificant, t-test. GAPDH was used as an endogenous loading control. Representative images from at least three independent experiments are shown.

We detected the expression of all LMN A/C forms in the mouse oocytes (Figs 1A and 3). Recently we reported that both LMN A and LMN C are expressed equally during meiotic progression in adult females, in particular between young (2 months old) and aged (12 months old) female groups [20]. Based on this, we analysed the expression of LMN C2 in the oocytes isolated from young and aged females during meiotic maturation from the GV to the MII stage. We found that expression of LMN C2 gradually decreases as oocytes progress in meiosis I (Fig 5A and 5B). Taking LMN C2 levels in GV oocytes of young females as 100%, expression of LMN C2 gradually decreased to 28% after 12h of meiotic maturation (metaphase II stage) in young female oocytes. Unexpectedly, we also found that the protein levels of LMN C2 are significantly decreased (P<0.05) in oocytes from aged females (Fig 5A and 5B). In oocytes of aged females, LMN C2 levels at GV stage were already only 21% of the levels in corresponding GV stage of young female oocytes. Expression of GAPDH was used as a loading control marker and despite the significant decrease of LMN C2 during meiotic progression and between the two age groups, there were no significant differences in GAPDH protein levels (Fig 5A), demonstrating that the detected decrease is specific for LMN C2.

Our results show that amount of the LMN C2 protein significantly decreases when oocytes resume meiosis and its expression significantly differs between oocytes derived from young and aged females.

### LMN C2 is expressed in the oocytes of different mammalian species

**Fig 6:**
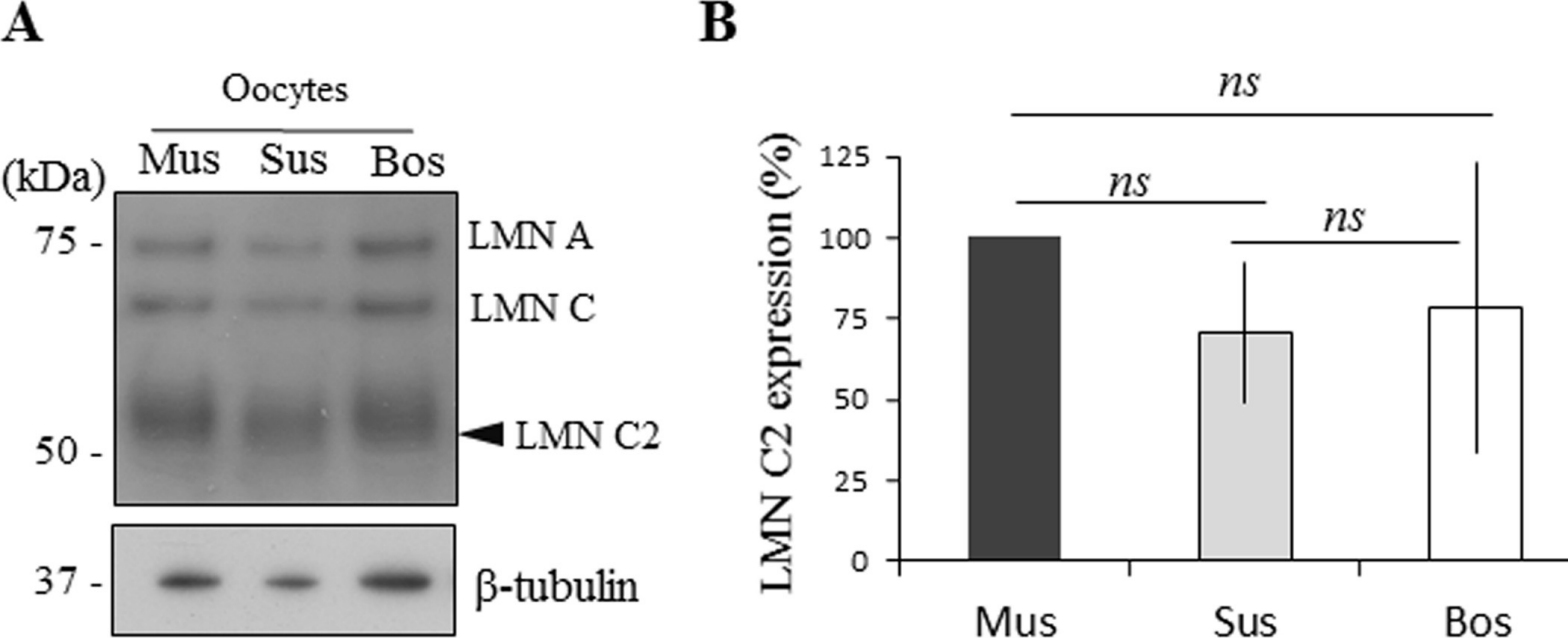
LMN C2 is expressed in the oocytes of different mammalian species. A) The detection of LMN A/C in the oocytes from mouse (Mus), pig (Sus) and cow (Bos). Black arrowhead depicts endogenous LMN C2 protein. β-tubulin was used as a loading control for all species tested. Representative image from three independent experiments. B) Quantification of LMN C2 protein expression in three mammalian species. Presented as mean, ±SD, ns-nonsignificant.

The expression of LMN C2 in the mouse oocyte has previously been reported. So we next asked, whether the LMN C2 protein is also expressed in other mammalian species. To address this, we performed immunoblotting on mouse, porcine and bovine oocytes. We detected all three LMN A/C forms including LMN C2 in the oocytes from all tested species (Fig 6A and 6B). Comparison of the expression levels of LMN C2 between mouse, porcine and bovine oocytes revealed no significant differences, suggesting that LMN C2 expression is almost the same in each. To standardize expression levels, we used β-tubulin as a loading control.

With this experiment we demonstrated that the LMN C2 protein is expressed in oocytes from all three tested species: mouse, pig and cattle.

## Discussion

In the current study we address the question of the expression of A-type lamins, with a particular focus on the expression of the short A-type isoform lamin C2, in the oocytes of mice, pigs and cattle. Lamin C2 is a short splice variant of the *Lmna* gene which was found to be selectively expressed during the meiotic stages of spermatogenesis (i.e. in spermatocytes) [7,9,10] and in embryonic oocytes [14]. Here we show that LMN C2 is also present in postnatal female germ cells i.e. the meiosis I arrested dictyate and the growing oocytes, in the protein form, whereas the corresponding mRNA appears to be absent at the later, adult stages of oocyte development. Our results suggest that *Lmnc2* mRNA is expressed and translated during the embryonic oocyte growth stage where LMN C2 might have a role in chromosome pairing and meiotic recombination [14]. During meiotic prophase I, LMN C2 localizes to the nuclear envelope and forms punctate structures at the sites where telomeres of the chromosomes attach [13]. In a LMN C2 deficient background, both oocytes and spermatocytes show defects in synaptic pairing of chromosomes, but in the spermatocytes these defects are more severe and frequent. Thus, LMN C2 deficiency shows sexual dimorphic pathology [14]. Despite this and given that in oocytes LMN C2 is still present at the nuclear envelope after synaptic pairing, it would be conceivable that during oogenesis the LMN C2 protein may have a second, subsequent function in later oocyte stages. Several studies demonstrated that during aging cells undergo age-related changes in nuclear architecture [20,23–25]. Accordingly, decreasing concentrations or the complete loss of LMN C2 protein may possibly lead to increased chromosomal segregation errors in oocytes from aged females. Similarly, levels of the meiotic cohesin protein REC8 are severely reduced on chromosomes in oocytes from aged mice which in turn contributes to an increase in aneuploidy [26,27]. The nuclear envelope plays a critical role in the regulation of many cellular events as well as maintaining genomic stability and regulating protein/RNA transport in and out of the nucleus [28,29]. Previously we reported that the expression of both LMN A and C does not change in oocytes with increased female age [20]. Surprisingly we found that the LMN C2 protein is significantly decreased in the oocytes of aged females in comparison to young females. Recently we also discovered that GV oocytes from female mice of advanced age have an aberrantly formed nuclear envelope [20], which strongly resembles the morphology of those in aged somatic cells [23,24]. von Glasenapp and Benavente (2000) suggest that degradation of LMN C2 at the onset of meiosis represents a critical step in the process of nuclear envelope disassembly. Taking this observation into account, it is tempting to speculate that a decreasing level of LMN C2 protein during aging may at least partly account for the abnormal nuclear membrane morphology observed in the oocytes from aged females [20]. In addition to this, with a reduced amount of LMN C2 the de-polymerization of nuclear membrane structures during nuclear envelope breakdown (NEBD) in oocytes from aged females may occur faster than in younger females [20]. Decreased levels of LMN C2 could further contribute to precocious NEBD in oocytes from aged females and thus the age-dependent decline of LMN C2 in oocytes could be a critical factor for female age-related fertility decline and reduced oocyte/embryo competence [20,30]. In a previous study it was observed that LMN C2 deficient female mice do not show significant decreased fertility rates, suggesting that the absence of LMN C2 may have only minor effects on oocyte development and maturation [14]. However, up to now direct age dependent fertility effects have not been studied in the LMN C2 deficient mouse line and thus this aspect remains still open.

The fact that *Lmnc2* mRNA is not present in adult oocytes while the corresponding protein is verifiable in the same oocyte stages is striking. This implies that the LMN C2 protein is produced during embryonic prophase I and then, at least to some extent, protected from degradation so that the protein is still detectable in the adult, dictyate arrested oocyte stages and even in the growing oocytes after natural hormonal induction [31] and fully grown oocyte. However, as shown here, the amount of LMN C2 protein is significantly decreasing after resumption of meiosis. The absence of LMN C2 mRNA and the elimination of the protein during meiosis I indicate that LMN C2 is not necessary for the terminal stages of meiosis and for early embryo development. This is consistent with the previous finding that LMN C2 deficient females are fertile and produce offspring [14]. Lamins are essential components of the nuclear envelope of eukaryotic cells. Depolymerization of the nuclear lamina structure at the end of prophase is a key event during the breakdown of the nuclear envelope which is governed by MPF as shown in a variety of cell types and oocyte [20,32,33]. Due to the absence of the phosphorylation site that is essential for lamina depolymerization in somatic A-type lamins, it is reasonable to assume that lamin C2 is not a substrate of MPF governed kinases. The possible elimination of LMN C2 in the oocyte post NEBD might by coupled with the dissociation of nuclear lamina.

Our results presented in this study provide evidence of the presence of LMN C2 at the nuclear membrane of oocytes, which disappears as the oocytes complete their first meiotic division. Furthermore, we demonstrated that the concentration of LMN C2 is significantly higher in the oocytes of younger females than in aged females, which could serve as partial explanation for the commonly observed multifactorial phenomenon of the age-related decrease in quality of oocytes.

## Materials and methods

### Isolation and preparation of mouse tissue, oocytes and testes

GV oocytes were obtained from CD1 mice 46 hours after stimulation by 5 IU pregnant mare serum gonadotropin (PMSG, HOR 272, ProSpec, Rehovot, Israel). The *Lmnc2*^*+/+*^ and *Lmnc2*^*-/-*^ samples used in this study were derived from the previously described *Lmnc2* specific mouse line Lmna^tm1Rben^ [14]. Ovaries of the *Lmnc2* mouse line were dissected from 11d old females, fixed in 1% formaldehyde in phosphate buffer saline (PBS) (pH 7.4) for 3 h and embedded in paraffin according to previous protocols [34]. Porcine and bovine ovaries were provided by a slaughterhouse and the oocytes were aspirated by syringe from follicles and frozen at the germinal vesicle (GV) stage. Juvenile ovaries were isolated from mice 1 day after birth. Livers were isolated also from 1 day old mice and after lysis were used for immunoblotting. All animal work was conducted according to Act No 246/1992 for the protection of animals against cruelty; from 25.09.2014 number CZ02389, issued by the Ministry of Agriculture.

Oocytes from young females were isolated from 2 month old mice and aged (preovulatory) oocytes from 12 month old mice. Oocytes were isolated at the germinal vesicle stage (GV; 0 h) in transfer medium [35] supplemented with 100 μM 3-isobutyl-1-methylxanthine (IBMX, I5879, Sigma-Aldrich, Darmstadt, Germany) to prevent NEBD. Selected fully grown GV oocytes were denuded by pipetting and cultured in M16 medium (M7292, Sigma-Aldrich) without IBMX at 37°C, 5% CO2. Post-IBMX-wash (PIW) oocytes underwent nuclear envelope breakdown (NEBD) within 1 h and oocytes arrested in GV stage were discarded. Oocytes undergoing maturation for 6 h PIW reached metaphase I and after 12 h metaphase II. For oocyte treatment, 10 μM cycloheximide (Sigma Aldrich) was added 2 h PIW and the oocytes then incubated another 5 h with cycloheximide. Testes were isolated from 4 week old males after cervical dislocation. Spermatogenic cells were isolated from 15 day old males by lysing seminiferous tubules in 0.1% trypsin in PBS with 20 μg/ml DNAse. 14 day old testes are composed of approximately 6% A type spermatogonia, 6% B spermatogonia, 9% preleptotene spermatocytes, 13% leptotene spermatocytes, 14% zygotene spermatocytes, 15% pachytene spermatocytes and 37% Sertoli cells [36].

Testes were used for RT-PCR and for immunoblotting whereas spermatogenic cells were used just for immunoblotting. Cumulus cells were isolated from cumulus enclosed oocytes after stripping by centrifugation in culture media. Testes from *Lmnc2* WT, KO and HZ males were lysed by lysis buffer composed of 7M urea, 2M thiourea, 3% CHAPS, 2% NP-40, 5mM TCEP with the addition of protease and phosphatase inhibitor. Testes were incised then incubated in lysis buffer 1 h at 4°C on vortex. Lysate was sonicated for 10 min and centrifuged at 20,000 g for 15 min at 4°C and supernatant collected. Samples were diluted in sample buffer and used for WB.

GV oocytes were microinjected in the presence of the IBMX on an inverted microscope Leica DMI 6000B (Leica Microsystems, Wetzlar, Germany) using TransferMan NK2 (Eppendorf, Hamburg, Germany) and FemtoJet (Eppendorf). Oocytes were injected with 20 ng/μl of plasmid LMNC2:DDK (Origine), 40 ng/μl of plasmid LMN C2:EGFP (developed by [12]) diluted in RNAse free water. Approximately 5 pL of solution was injected into each oocyte. 2 h after injection the oocytes were scanned in cover-glass-based 4-well chambers (94.6190.402, Sarstedt, Nümbrecht, Germany) in a Leica SP5 inverted confocal microscope (Leica Microsystems, Wetzlar, Germany). Oocytes microinjected with plasmid were incubated 14 h before freezing or fixation by 4% paraformaldehyde. Microinjected oocytes were used for immunoblotting and immunocytochemistry.

All animal work was conducted according to Act No 246/1992 for the protection of animals against cruelty; from 25.09.2014 number CZ02389, issued by the Ministry of Agriculture (experimental project #215/2011).

### Immunoblotting

Oocytes were washed in PBS (Sigma-Aldrich) with polyvinyl alcohol (PVA, Sigma-Aldrich) and frozen to -80°C. An exact number of oocytes (15–30) and small piece of liver were lysed in 10 μL of 1x Reducing LDS Loading Buffer (lithium dodecyl sulfate sample buffer NP 0007 and reduction buffer NP 0004, Thermo Fisher Scientific, Waltham, MA, USA) and heated at 100°C for 5 min. Proteins were separated by gradient precast 4–12% SDS–PAGE gel (NP 0323, Thermo Fisher Scientific) and transferred to Immobilon P membrane (IPVD 00010, Millipore, Merck group, Darmstadt, Germany) using a semidry blotting system (Biometra GmbH, Analytik Jena, Jena, Germany) for 25 min at 5 mA/cm^-2^. Membranes were blocked by 5% skimmed milk dissolved in 0.05% Tween-Tris buffer saline (TTBS), pH 7.4 for 1 h. After a brief wash in TTBS, membranes were incubated at 4°C overnight with the primary antibodies lamin A/C (mouse, SAB4200239, Sigma Aldrich), β-Tubulin (Rabbit, 2128, Cell Signalling) and GAPDH (rabbit, G9545, Sigma Aldrich) diluted 1:7500 in 1% milk/TTBS. Secondary antibody Peroxidase Anti-Rabbit Donkey (711-035-152) or Peroxidase Anti-Mouse Donkey (715-035-151, Jackson ImmunoResearch, West Grove, PA, USA) was diluted 1:7500 in 1% milk/TTBS. The membranes were incubated in the secondary antibodies for 1 h at room temperature. Immunodetected proteins were visualized on films using ECL (Amersham, GE Healthcare Life Sciences, Barcelona, Spain). Films were scanned using a GS-800 calibrated densitometer (Bio-Rad Laboratories, CA, USA) and quantified using ImageJ (http://rsbweb.nih.gov/ij/). Full immunoblots of segments shown in the main figures are shown in S2 and S3 Figs.

### Immunocytochemistry

Microinjected oocytes 14 h after injection were fixed for 15 min in 4% paraformaldehyde (PFA, Alfa Aesar, Thermo Fisher Scientific, Waltham, MA, USA) in PBS. Oocytes were permeabilized in 0.1% Triton (X-100, Sigma-Aldrich) in PBS/PVA for 10 min, washed in PBS/PVA, and incubated overnight at 4°C with primary antibody lamin A/C (mouse, SAB4200239, Sigma Aldrich). After washing in PBS/PVA, detection of the primary antibodies was performed by cultivation of the oocytes with relevant Highly Cross-Adsorbed Secondary Antibody, Alexa Fluor 594 conjugate (Thermo Fisher Scientific) diluted 1:250 for 1 h at room temperature. Oocytes were then washed two times for 15 min in PBS/PVA and mounted using a Vectashield Mounting Medium with DAPI (H-1200, Vector Laboratories, Burlingame, CA, USA). Samples were visualized using a Leica SP5 inverted confocal microscope (Leica Microsystems, Wetzlar, Germany). Images were assembled in software LAS X (Leica Microsystems).

Paraffin sections (3 μm) of ovaries of 11d old WT or LMN C2 –KO mice, were immunostained with #bs-01, LMN A/C antibody (developed by: [17]) and guinea pig anti-SYCP3 antibody (developed by: [9]) and counterstained for DNA using Hoechst (33258, Sigma-Aldrich).

### RNA Isolation and RT-PCR

RNA from juvenile ovaries, oocytes and testes were extracted using an RNeasy Plus Micro kit (74034, Qiagen, Hilden, Germany) and genomic DNA was removed using gDNA Eliminator columns. For reverse transcription was used qPCRBIO cDNA Synthesis Kit (PB30.11, PCR Biosystems, London, UK) with reaction condition: 42°C/30 min and denaturation of RTase at 85°C/10 min. RT-PCR was then carried out using the QuantStudio®3 and the Luna® Universal qPCR Master Mix (M3003, New England BioLabs, Ipswich, MA, USA) according to manufacturer’s protocols. *Lmnc2* specific RT-PCR primers (Forward: GAACTCCTGAGGGCGCAAG, Reverse: ACGCAGTTCCTCGCTGTAAA) and *Gapdh* (Forward: TGGAGAAACCTGCCAAGTATG, Reverse: GGTCCTCAGTGTAGCCCAAG) were designed with an annealing temperature of 60°C. The reaction condition was for initial denaturation at 95°C/60 sec, followed by 40 PCR amplification cycles (95°C/15 s, 60°C/30 s) and melt curve 60–95°C/1 min with 1.6°C/s increase. Products were visualized by gel electrophoresis on 1.5% agarose gel with gel red as marker using 100 bp plus DNA ladder (SM0321, Thermo Fisher Scientific). Full gel of segment shown in the main figure is shown in S2 Fig.

### Statistical analysis

Mean and standard deviation (SD) values were calculated using MS Excel. Statistical significance of the differences between the groups was tested using Student’s t-test (PrismaGraph5) and p <0.05 was considered as statistically significant (marked by asterisks: * p < 0.05; ** p < 0.01; *** p < 0.001).

## Supporting information

S1 Fig Cycloheximide (CHX) treatment suppresses global protein synthesis in the oocytes. To validate translational repression by CHX we treated GV oocytes by 10 μg/ml of CHX for 2hrs in the presence of global translation marker ^35^S-Methionine. GAPDH was used as an endogenous loading control.
(TIF)

S2 Fig Full immunoblots and PCR gels of segments shown in the main Figs 1 and 4A. Rectangle denote the bands presented.
(TIF)

S3 Fig Full immunoblots and PCR gels of segments shown in the main Figs 4B, 5, 6 and S1. Rectangle denote the bands presented.
(TIF)
