# Supplementary figures and images for: Expression of lamin C2 in mammalian oocytes
Source: PLoS One. 2020 Apr 28;15(4):e0229781. doi: 10.1371/journal.pone.0229781 (PMC7188254; doi:10.1371/journal.pone.0229781)

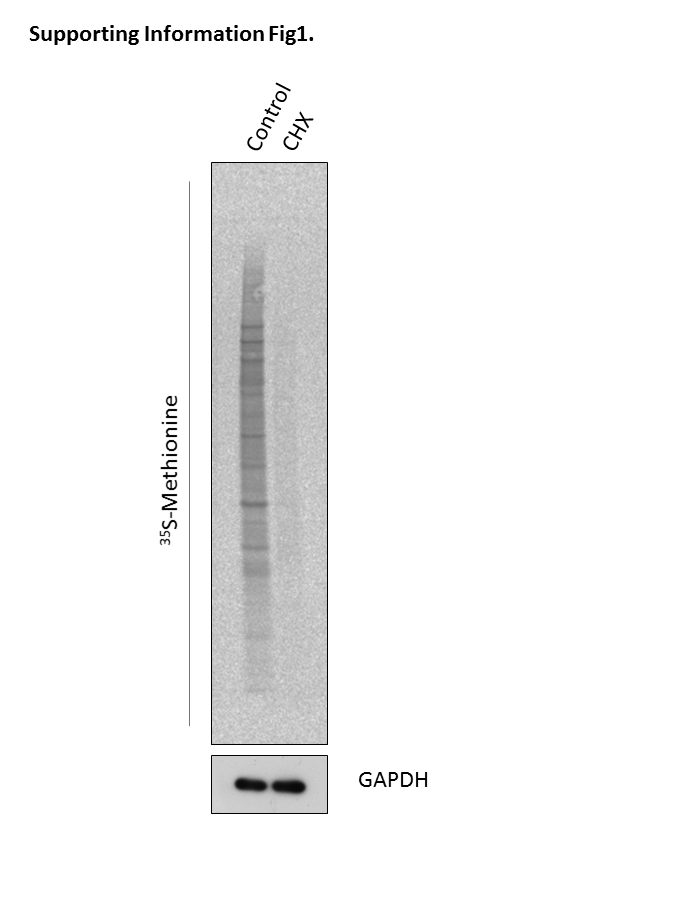

Supplement: S1 Fig — To validate translational repression by CHX we treated GV oocytes by 10 μg/ml of CHX for 2hrs in the presence of global translation marker 35S-Methionine. GAPDH was used as an endogenous loading control. (TIF) [file pone.0229781.s001.TIF]

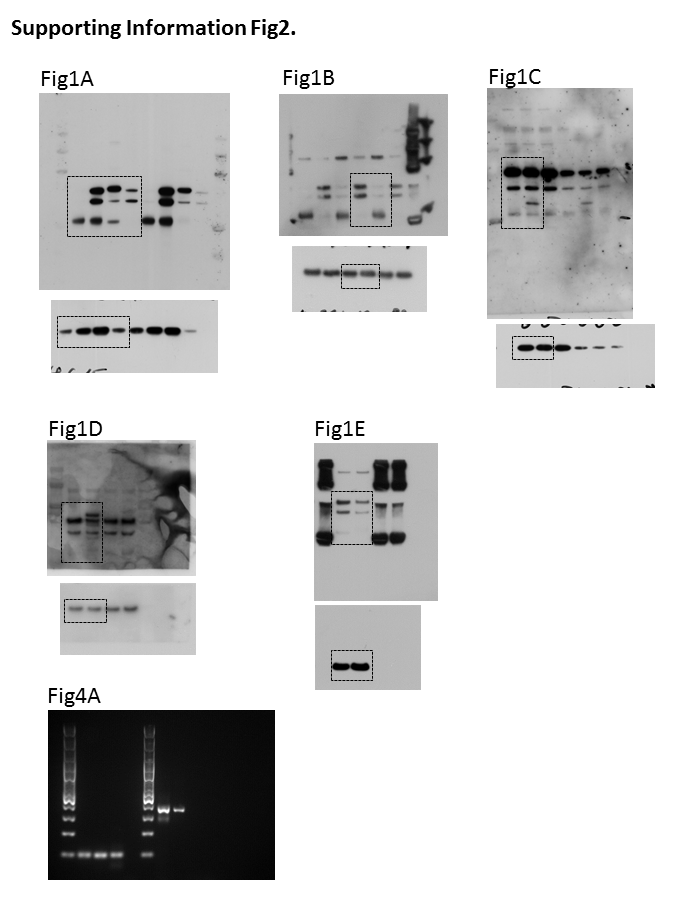

Supplement: S2 Fig — Rectangle denote the bands presented. (TIF) [file pone.0229781.s002.TIF]

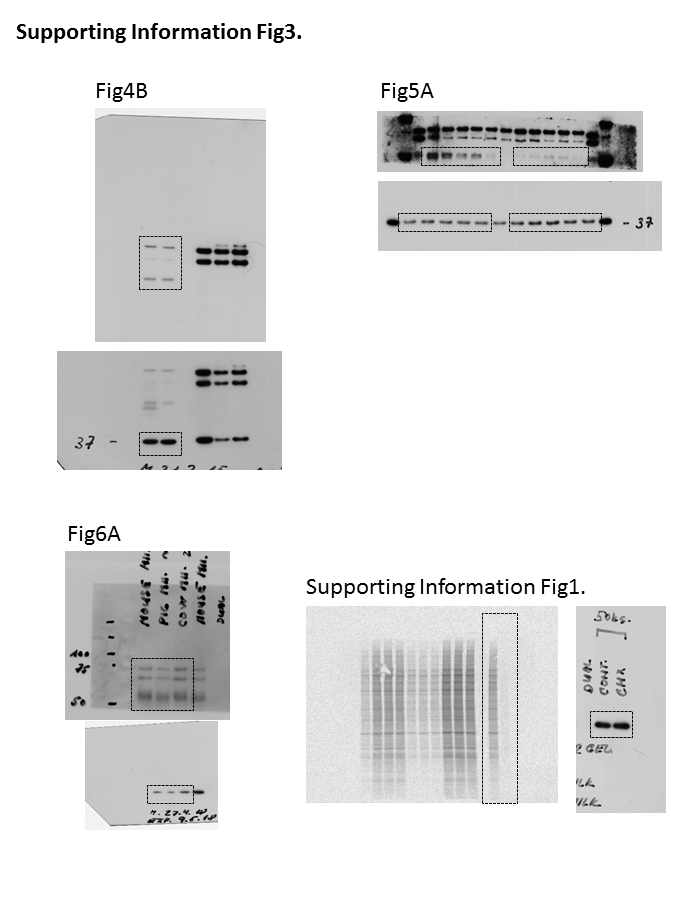

Supplement: S3 Fig — Rectangle denote the bands presented. (TIF) [file pone.0229781.s003.TIF]
